# Endodontic Retreatment Using Rotary System and Aesthetic Rehabilitation of a Deciduous Central Maxillary Incisor in a 5‐Year‐Old Child: A Case Report

**DOI:** 10.1155/crid/5510372

**Published:** 2026-06-04

**Authors:** Esthefany Domínguez-Gaibor, Leslee Ribadeneira-Morales, Paulina Sempertegui, Juan Marcos Parise-Vasco

**Affiliations:** ^1^ Postgraduate Program in Pediatric Dentistry, Faculty of Health Sciences Eugenio Espejo, Universidad UTE, Quito, Ecuador, ute.edu.ec; ^2^ School of Health Sciences and Human Well-Being, Universidad Tecnológica Indoamérica, Quito, Ecuador

**Keywords:** aesthetic restorations, case report, celluloid crowns, early childhood caries, pediatric endodontics, pulp retreatment

## Abstract

**Introduction:**

Early childhood caries (ECC) affects the primary dentition of preschool children and can result in the need for pulp treatment. Endodontic retreatment in deciduous teeth is indicated when initial treatment fails, representing a conservative alternative to extraction.

**Objective:**

This case describes pulp retreatment using a rotary system and complete aesthetic rehabilitation of the anterosuperior sector in a pediatric patient.

**Case Report:**

A 5‐year‐old systemically healthy male patient attended the clinic with ECC affecting the maxillary central and lateral incisors. Clinical examination revealed a temporary dentition with bilateral Class I molar relationship. The left maxillary central incisor presented a previous deficient endodontic obturation with material extending only one‐third of the root length. The treatment plan included pulp retreatment of Tooth 61, fiberglass postplacement, and celluloid crowns on maxillary incisors, in addition to comprehensive oral cavity rehabilitation. Canal debonding was performed with K‐files, removing zinc oxide eugenol. Reinstrumentation was performed with Hedstrom File #30 and the U1 file rotary system (0.40 mm, torque 2.5 N·m, and 400 RPM), with a working length of 11.5 mm. An irrigation protocol was performed using physiological saline, as well as 2.5% and 5% sodium hypochlorite. Obturation was performed with Metapex. Subsequently, a fiberglass post cemented with RelyX dual and celluloid crown with Filtek Supreme resin were placed. One‐year follow‐up demonstrated an asymptomatic tooth, remaining in the mouth in favorable conditions with successful aesthetic and functional restoration. The mother and patient expressed high satisfaction with the outcome.

**Conclusion:**

In this pediatric case, endodontic retreatment of a deciduous maxillary central incisor using a rotary system, combined with fiberglass post placement and celluloid crown rehabilitation, resulted in favorable clinical and radiographic outcomes at 12‐month follow‐up. Within the limitations of a single‐case report, these findings suggest that retreatment may be considered a viable conservative alternative to extraction in carefully selected clinical scenarios.

## 1. Introduction

Early childhood caries (ECC) is a severe form of tooth decay that affects the primary dentition of preschool age children. It has a multifactorial origin, involving various biological, behavioral, and environmental factors. [1, 2] It has been commonly defined as the presence of one or more primary teeth with carious lesions, missing, or treated with fillings in children aged 71 months (5 years and 11 months) or younger. [3] Contributing factors include feeding habits, including prolonged breastfeeding associated with frequent bottle use and habitual consumption of sugary foods or beverages. Additionally, there are structural enamel defects and low‐income groups, among others. [4–6]

The prevalence of ECC has been widely studied in various investigations. Several studies conclude that a lower level of parental education is associated with an increased risk of developing ECC in preschool children over a 24‐month period. [7] Likewise, factors such as the presence of pre‐existing caries, biofilm accumulation, and higher *Streptococcus mutans* counts have been found to significantly increase the predisposition to the development of new carious lesions within the same period. [8] Lack of timely care in children can result in a series of consequences, such as infections, aesthetic problems, feeding difficulties, language disorders, and the appearance of malocclusions. Additionally, it can have medical, emotional, and financial repercussions. ECC is considered a risk factor for future caries development in both the primary and permanent dentition. [2, 3]

Adequate oral health care during the primary dentition stage is essential to promote overall well‐being in children. Repairing teeth that have been severely damaged by irreversible pulp compromise is challenging for pediatric dentists. They must consider three fundamental aspects: managing patient behavior, preserving dental structure and ensuring parental satisfaction. [9] Various options are available to treat dental caries lesions among pediatric patients. Preventive treatments include fluoride application, atraumatic restorative technique, and sealant application. For cases where caries has affected the dental pulp, pulp treatments such as pulpotomy, pulpectomy, and indirect pulp capping can be performed. [3, 10] Pulpectomy is an endodontic treatment indicated in primary teeth with irreversible pulp involvement caused by extensive caries or trauma. [11, 12] When pulpectomy is not feasible due to advanced root resorption, loss of supporting structure, compromised remaining root length or an unfavorable relationship with the permanent tooth germ, extraction of the affected primary tooth followed by placement of an aesthetic fixed space maintainer is a valid alternative, preserving arch length and supporting aesthetic and functional demands until the permanent successor erupts. [13–15]

However, in some cases, initial treatment may fail, making pulpectomy retreatment necessary. [16] This procedure is indicated when signs of endodontic failure are present, such as persistent pain or prolonged sensitivity, recurrent abscesses or fistulas, increasing periapical radiolucency, accelerated or pathological root resorption, and defects in canal obturation, either due to underfilling or overfilling. [17] Despite available evidence on primary pulpectomies in deciduous teeth, there is limited and scattered information on selection criteria, specific techniques, and prognosis of endodontic retreatment in temporary dentition, particularly when rotary instrumentation is employed. [18, 19]

Regarding restorative treatments for deciduous anterior teeth, stainless steel crowns, transparent and colored polycarbonate crowns, preformed zirconia crowns, as well as improved composite resin crowns manufactured in laboratory and celluloid crowns can be used. [20] It is important that a pediatric dentist evaluates each case and determines the most appropriate treatment for each specific situation. [20, 21] Integration of endodontic retreatment with prosthetic rehabilitation using fiber posts and aesthetic crowns requires greater clinical documentation to guide therapeutic decision‐making. [22]

The aim of this article is to report on the retreatment and comprehensive rehabilitation of the anterior deciduous teeth of a 5‐year‐old patient, providing a detailed description of the technical protocol employed.

## 2. Case Report

**Figure 1:**
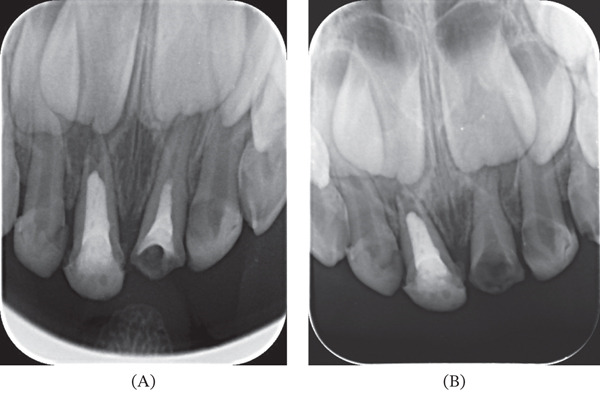
Initial periapical radiographs. (A) Initial radiograph and (B) canal disobliteration.

A 5‐year‐old systemically healthy and asymptomatic male patient (5 years and 1 month of age) attended the UTE university clinic accompanied by his caregiver. No known allergies or current medications were recorded. The patient did not have a relevant medical history that contraindicated dental treatment. During oral exploration, a complete temporary dentition with bilateral Class I Angle molar relationship was observed. Caries were detected in almost all of the teeth, with the exception of the right maxillary canine (53), the right mandibular canine (83), and the left mandibular canine (73). Periapical radiographs of the anterior sector were performed. Radiographic examination of Tooth 61 revealed a previous root canal obturation with radiopaque material compatible with zinc oxide eugenol, reaching only one‐third of the root length, constituting a deficient obturation (Figure 1). Although the tooth was clinically asymptomatic, with no signs of fistula, abscess, pathological mobility, or tenderness on percussion, the radiographic finding of an underfilled canal was considered a risk factor for future endodontic failure and reinfection. The decision to perform retreatment rather than extraction was based on a combination of clinical and radiographic criteria: the absence of advanced pathological root resorption, integrity of the lamina dura and supporting periodontal structures, sufficient remaining root length, and a favorable relationship with the developing permanent tooth germ. These findings supported the feasibility of a conservative approach aimed at retaining the primary tooth until natural exfoliation. The clinical and radiographic findings, as well as the therapeutic alternatives including extraction with placement of an aesthetic fixed space maintainer were discussed with the patient′s legal guardians, who gave informed consent to the proposed treatment plan.

The comprehensive treatment plan included sealants on the second maxillary molars and the right second mandibular molar, restorations on the first maxillary molars, the left second mandibular molar, and the central and lateral mandibular incisors, as well as pulp therapy followed by the placement of stainless‐steel crowns on the first mandibular molars. The left upper primary central incisor required pulpal retreatment and placement of a fiber post with subsequent restoration using a celluloid crown. In addition, celluloid crowns were planned for the central and lateral maxillary incisors to enhance aesthetics.

Treatment began with oral hygiene instruction provided to the caregiver and the child, emphasizing the importance of proper hygiene to ensure the long‐term success of oral rehabilitation. The patient was taught the Fones brushing technique. Plaque disclosure, dental prophylaxis, and topical fluoride application with 5% sodium fluoride varnish (Climpro White Varnish, 3M ESPE) were performed.

Then pulpal retreatment of the primary central incisor of the left maxillary was initiated. Carious tissue and pre‐existing obturation material, consisting of zinc oxide–eugenol, were removed. Initial canal instrumentation was performed using first‐series K‐files (Dentsply Sirona Maillefer, Switzerland) under irrigation with a saline solution. A second instrumentation phase followed, beginning with a #30 Hedström file to a previously established working length of 11.5 mm determined using an apex locator. The irrigation was performed with 2.5% sodium hypochlorite. A rotary U1 file system (0.40 mm, Training Kit, United States) was then used at 2.5‐N·m torque and 400 RPM for 2 min, followed by a final irrigation using 5% sodium hypochlorite. The canal was subsequently obturated using a calcium hydroxide, iodoform, and oily silicone paste (Metapex, South Korea). A cotton pellet was placed, and the access cavity was sealed with composite resin. Radiographic evaluation demonstrated adequate obturation and sealing.

**Figure 2:**
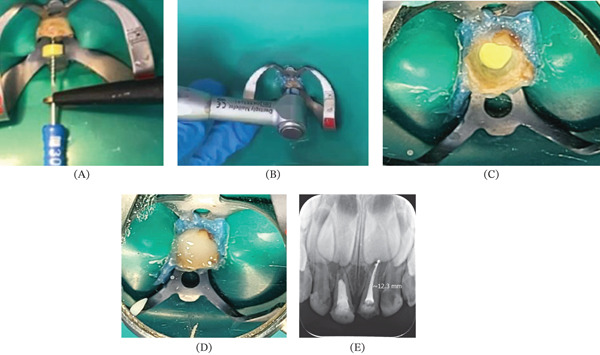
Steps of the pulpal retreatment. (A) Reinstrumentation, (B) rotary system, (C) obturation, (D) resin sealing, and (E) final obturation

At the following appointment, the coronal access was reopened and both cotton pellet and resin that had been placed were removed. Approximately one‐third of the canal filling was mechanically removed with a curette to create space for the fiber post. The length of the post was determined by placing the post inside the canal and marking the required height, after which it was cut using a cylindrical diamond bur (1090) mounted on a high‐speed handpiece. The post was disinfected with 70% isopropyl alcohol and treated with silane (Angelus, Brazil) for 1 min. Adper Single Bond (3M, United States) was applied and light‐cured for 20 s. In parallel, the canal and pulp chamber were conditioned with 37% phosphoric acid for 20 s and rinsed for 40 s. Excess moisture was gently removed with cotton pellets before applying a seventh‐generation adhesive, which was photo‐cured for 20 s (Figure 2).

**Figure 3:**
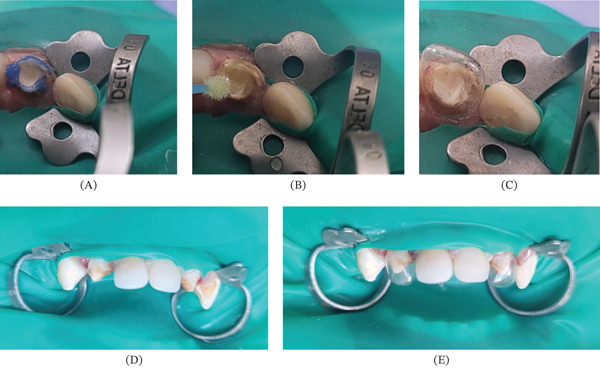
Placement of celluloid crowns. (A) Application of phosphoric acid, (B) application of the bonding agent, (C) celluloid crown implantation, (D) aesthetic restoration of the central maxillary incisors, and (E) celluloid crown implantation for the lateral maxillary incisors.

Dual‐cure resin cement (RelyX, 3M, United States) was applied inside the canal and on the fiber post, which was carefully inserted, allowing excess cement to flow out before light curing. The tooth was restored using a celluloid crown filled with Filtek Supreme composite resin (3M, United States), achieving optimal esthetic and functional adaptation. Restorations previously placed on the lateral maxillary incisors and the central right maxillary incisor were removed due to marginal leakage, and these teeth were rehabilitated with celluloid crowns to enhance aesthetics. Then complete rehabilitation of the remaining carious teeth was performed, leaving the patient free of active lesions (Figure 3).

**Figure 4:**
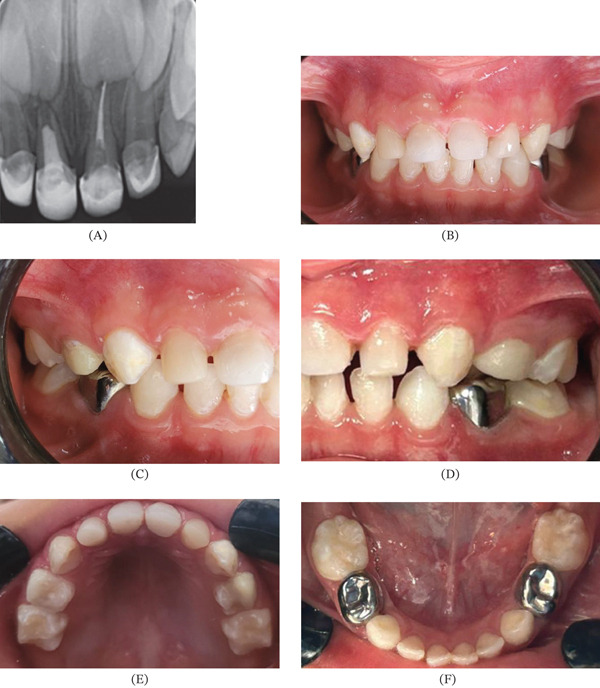
Final results. (A) Final radiograph, (B) frontal view, (C) right lateral view, (D) left lateral view, (E) maxillary arch, and (F) mandibular arch.

The final result showed that the central and lateral maxillary incisors had been successfully restored in terms of both function and aesthetics, and that the oral cavity had been comprehensively rehabilitated. The retreatment of Tooth 61 represented a clinical challenge due to the need to remove the previous obturation material and to address the extensive carious involvement; however, 1‐year follow‐up confirmed that the tooth remained clinically and radiographically stable and retained in the arch. The caregiver expressed high satisfaction with the aesthetic results, highlighting the natural color and shape of the rehabilitated anterior teeth (Figure 4). Following complete rehabilitation, the child showed a positive attitude, as evidenced by increased confidence when smiling and speaking in social situations. Parents expressed great satisfaction with the outcome of the aesthetic treatment, specifically highlighting the naturalness of the color and the aesthetic form of the rehabilitated teeth. The aesthetic restoration of the anterior sector provided parents with peace of mind regarding their child′s oral health and appearance.

## 3. Ethical Considerations

This case report was conducted in accordance with the ethical principles established by the Declaration of Helsinki and its subsequent amendments. Prior to the initiation of treatment and data collection, the patient′s legal guardian signed the informed consent form and agreed to the treatment plan. The guardian also agreed to the use of patient clinical information and photographs, which will be presented anonymously for publication in a scientific journal. No procedure was performed beyond the scope of conventional pediatric dental care, and the treatment plan was designed exclusively for the therapeutic benefit of the patient.

## 4. Discussion

This clinical case documents the successful endodontic retreatment of a deciduous central maxillary incisor (Tooth 61) that presented previous pulpectomy failure manifested by deficient obturation extending only one‐third of the root length. The therapeutic approach integrated the use of a rotary system (U1 file: 0.40 mm) for canal reinstrumentation, followed by prosthetic rehabilitation using a fiberglass post and celluloid crown, complemented with comprehensive aesthetic rehabilitation of the anterosuperior sector. Clinical and radiographic follow‐up at 12 months demonstrated tooth permanence in the mouth in asymptomatic and functionally favorable conditions, with high satisfaction reported by both the patient and his family.

Pulp retreatment in deciduous teeth presents itself as a conservative therapeutic alternative to extraction, allowing prolonged retention of the temporary tooth until natural exfoliation. In this clinical case, the decision to perform retreatment on Tooth 61 was based on fulfillment of specific favorable clinical criteria: absence of advanced root resorption, lamina dura integrity, adequate infection control, and sufficient remaining root length (11.5‐mm working length) that allowed effective apical sealing without compromising permanent germ development. In contrast to extraction followed by an aesthetic fixed space maintainer, retreatment was considered preferable in this case, as the favorable clinical criteria enabled the preservation of the natural tooth, which offers biological, functional, and developmental advantages over prosthetic space management.

During the procedure, it was identified that the previous obturation material was zinc oxide eugenol, which was carefully removed using K‐files without apparent damage to the developing permanent tooth or temporary tooth. The selection of appropriate materials and techniques is fundamental to minimizing the inherent risks of retreatment in deciduous dentition. [21] In this regard, radiographic evidence of an apparently incomplete root canal filling in a primary tooth previously treated with pulpectomy should be interpreted cautiously. Although this finding in the present case suggested a deficient prior obturation, some materials used in primary teeth, particularly zinc oxide–based pastes, may undergo intracanal degradation over time, creating an image of partial filling (tunnel effect) despite an initially acceptable procedure. [23, 24] Recognizing this possibility is clinically relevant, as it may affect the interpretation of radiographic findings and the decision between retreatment and observation in asymptomatic teeth. In this case, radiographic appearance, together with the overall clinical context, supported retreatment to reduce the risk of reinfection.

According to Silva et al., [18] pulp retreatment achieved 68.06% longevity. Teeth subjected to this procedure showed a notable increase in survival time. Depending on the odontogenesis stage, this increase in dental retention can be beneficial for post and crown placement, which would contribute to greater retention and tooth aesthetics. [18] When considering “dental retention” as a secondary outcome, a significant difference in dental retention time was observed between teeth receiving retreatment. [25] It was found that new treatment provided additional survival time of at least 8.3 months. [18] For their part, Brochado et al. indicates that retreatment not only offers a high success rate but also represents a less invasive and effective strategy with favorable long‐term results. [26] The present case aligns with these findings, demonstrating the survival of Tooth 61 at 12 months posttretreatment. However, it should be noted that the 12‐month follow‐up in this case is insufficient to make definitive comparisons with the longevity rates reported in studies involving larger samples and longer follow‐ups.

Despite the favorable results documented in this case, it is fundamental to recognize that endodontic retreatment in the deciduous dentition is not without controversies in the scientific literature. Riis et al. reported that endodontic retreatment involves certain risks, including greater removal of remaining dentin and the possible appearance of canal wall defects, increasing the probability of vertical root fractures and eventual tooth loss. [27] Additionally, this type of intervention can negatively affect the developing permanent tooth, generating possible malformations. Therefore, in multiple clinical situations, temporary tooth extraction is chosen rather than complex pulp procedures. [28] In this context, the decision between retreatment and extraction must be made on a case‐by‐case basis. The selection criteria used in this case—radiographic evaluation of root resorption, integrity of the supporting structure, remaining root length, and favorable relationship with the permanent germ—represent the determining factors that favored the conservative option. Adequate planning and rigorous clinical and radiographic follow‐up are essential elements of comprehensive pediatric dental treatment and can represent a valid and predictable option.

The use of U1 file rotary system (0.40 mm) with specific torque parameters (2.5 N·m) and speed (400 RPM) in this case allowed efficient reinstrumentation of the previously treated canal. The literature suggests that rotary systems may offer advantages over manual instrumentation, including greater speed, better canal centering, and less operator fatigue. In the pediatric context, reducing the operative time is particularly relevant given that patients of preschool age present limitations in their cooperation capacity and tolerance to prolonged procedures. However, specific evidence on the use of a rotary system in deciduous tooth retreatments remains limited in the scientific literature. [20, 21, 27, 28]

For restorations of the anterior deciduous sector, Almajed mentions several alternatives, including celluloid crowns, direct resin restorations, stainless steel crowns, zirconia crowns, and CAD/CAM crowns. [29, 30] In this clinical case, celluloid crowns were chosen due to their aesthetic characteristics, reduced clinical time, and high degree of satisfaction reported by parents.

Celluloid crowns are especially suitable for primary anterior teeth with extensive coronal destruction and have demonstrated a restoration success rate greater than 80% in prolonged clinical follow‐ups. [31] Various studies support celluloid crown use in anterior temporary teeth for their high retention rate and good aesthetics, [29] surpassing direct composite resin restorations. [32, 33]

Zirconia crowns stand out for superior aesthetic appearance and mechanical resistance but require greater tooth preparation and have a high cost that can limit accessibility. [34–36] Finally, CAD/CAM crowns such as Zirkid represent promising innovation, although robust scientific evidence is still needed to confirm their clinical efficacy and long‐term acceptance. [30] The choice of celluloid crowns in this case was based on a combination of factors including institutional availability, cost‐effectiveness, clinical time, ability to achieve immediate satisfactory aesthetic results, and operator familiarity with technique. It is important to recognize that this decision was based on practical considerations and clinical preference rather than direct comparative evidence of superiority over other prosthetic options for this specific case.

Fiberglass posts can be considered for anterior primary teeth with extensive coronal loss when adhesive retention alone is inadequate. [37] When limited to the coronal third of the canal, they may preserve the physiological resorption process while improving restorative retention. Their dentin‐like elasticity may also reduce stress transmission and the risk of root fracture. Fiberglass posts can be safely used in anterior primary teeth with extensive coronal loss, provided they are limited to the coronal portion of the canal, as this approach does not appear to interfere with the physiological resorption process or natural exfoliation. [38, 39] In the present case, the 12‐month follow‐up showed no clinical or radiographic signs of altered root resorption pattern, periapical pathology, or interference with the development of the permanent successor, supporting the biological compatibility of this approach in this specific clinical scenario.

Pediatric dentists often face the challenge of performing pulp treatments and restorations on anterior teeth, especially in the maxilla, where ECC commonly affects the incisors. [34, 40–42] This type of procedure can be challenging, as most cases involve children under 72 months of age, who tend to be less cooperative during treatment and failure of treatment can occur. [31] According to Zhang et al., early childhood dental caries is a common problem affecting the maxillary incisors, which usually have short and narrow crowns. [42] Therefore, a restorative technique is needed that allows effective, durable, functional, and aesthetically pleasing restorations in these teeth. [35]

The aesthetic rehabilitation of the anterior sector in pediatric patients transcends merely the restoration of dental function. In this case, the positive impact on the child′s self‐esteem was documented, evidenced by increased confidence when smiling and speaking in social situations. Parents expressed great satisfaction with the aesthetic result, highlighting the naturalness of the color and form of the rehabilitated teeth, providing peace of mind about their child′s oral health and appearance. This psychosocial aspect is particularly relevant in preschool age, the period in which children begin developing self‐awareness of their appearance and may experience negative effects on their self‐esteem due to dental aesthetic alterations.

One of the strengths of the report on this case is its documentation of the specific technique, including the materials used and quantifiable operational parameters such as torque (2.5 N·m), speed (400 RPM), and light curing time, as well as the adhesive‐cementing systems. This allows the protocol to be reproduced. The biomimetic approach is also evident in the selection of fiberglass posts with an elasticity modulus like dentin and dual‐curing cements. This approach prioritizes the preservation of remaining dental tissue and respects the biology of the deciduous tooth. However, one limitation is that, as this is a single case report without a control group, definitive causal relationships cannot be established, nor can the effectiveness of retreatment be compared with therapeutic alternatives. The results cannot be generalized to the pediatric population. Although 12‐month follow‐up is adequate to detect early complications, it is insufficient to evaluate longevity until natural exfoliation.

Prospective studies with larger samples, comparison groups, prolonged follow‐ups until natural exfoliation and permanent tooth eruption, and the use of validated outcome evaluation tools are required to establish with greater certainty the effectiveness, long‐term safety, and cost–benefit relationship of endodontic retreatment in deciduous dentition. Until such evidence is available, retreatment should be considered as an additional therapeutic option within the comprehensive management of deciduous teeth with endodontic failure, whose selection must be carefully individualized based on the specific characteristics of each clinical case.

## 5. Conclusions

In this 5‐year‐old pediatric patient, endodontic retreatment of the left deciduous maxillary central incisor with a rotary system, followed by prosthetic rehabilitation with a fiberglass post and celluloid crown, resulted in favorable clinical and radiographic outcomes at 12‐month follow‐up, with high satisfaction reported by the patient and caregiver. The detailed description of the technical protocol including irrigation regimen, rotary instrumentation parameters, adhesive cementation sequence, and prosthetic rehabilitation is the main contribution of this report and may serve as a reference for clinicians managing similar cases. Within the limitations inherent to a single‐case report, these findings suggest that retreatment may be considered a viable conservative alternative to extraction in carefully selected clinical scenarios with favorable anatomical and biological conditions.

## Author Contributions

**E.D-G.:** conceptualization, methodology, investigation (clinical execution of the treatment), data curation, resources, visualization, writing—original draft, and writing—review and editing. **L.R-M.:** methodology, validation, writing—original draft, and writing—review and editing. **P.S.:** conceptualization, methodology, investigation (clinical execution of the treatment), and writing—review and editing. **J.M.P-V.:** methodology, supervision, validation, writing—original draft, and writing—review and editing.

## Funding

No funding was received for this manuscript.

## Disclosure

All authors have read and approved the final version of the manuscript and agree to be accountable for all aspects of the work.

## Conflicts of Interest

The authors declare no conflicts of interest.

## Data Availability

Data sharing is not applicable to this article as no datasets were generated or analyzed during the current study.
